# Carbon Nanofibers and Their Composites: A Review of Synthesizing, Properties and Applications

**DOI:** 10.3390/ma7053919

**Published:** 2014-05-15

**Authors:** Lichao Feng, Ning Xie, Jing Zhong

**Affiliations:** 1 School of Mechanical Engineering, Huaihai Institute of Technology, Lianyungang 22205, Jiangsu, China; E-Mail: fenglichao33@163.com; 2 Harbin Institute of Technology, Harbin 150001, Heilongjiang, China; E-Mail: zhongjing.hit@gmail.com; 3 Jiangsu Marine Resources Development Research Institute, Lianyungang 22205, Jiangsu, China; 4 Research and Development Department, Lianyungang Zhongfu Lianzhong Composites Group Co., Ltd., Lianyungang 22206, Jiangsu, China

**Keywords:** carbon nanofibers, composites, dispersion, properties, percolation, fractal, modeling, batteries, sensors

## Abstract

Carbon nanofiber (CNF), as one of the most important members of carbon fibers, has been investigated in both fundamental scientific research and practical applications. CNF composites are able to be applied as promising materials in many fields, such as electrical devices, electrode materials for batteries and supercapacitors and as sensors. In these applications, the electrical conductivity is always the first priority need to be considered. In fact, the electrical property of CNF composites largely counts on the dispersion and percolation status of CNFs in matrix materials. In this review, the electrical transport phenomenon of CNF composites is systematically summarized based on percolation theory. The effects of the aspect ratio, percolation backbone structure and fractal characteristics of CNFs and the non-universality of the percolation critical exponents on the electrical properties are systematically reviewed. Apart from the electrical property, the thermal conductivity and mechanical properties of CNF composites are briefly reviewed, as well. In addition, the preparation methods of CNFs, including catalytic chemical vapor deposition growth and electrospinning, and the preparation methods of CNF composites, including the melt mixing and solution process, are briefly introduced. Finally, their applications as sensors and electrode materials are described in this review article.

## 1. Introduction

Since the first carbon fiber (CF), which was prepared by carbonizing cotton and bamboo, was used as the filament of a light bulb in 1879 by Thomas Edison, it has been developed tremendously in both fundamental scientific research and practical applications [1–5]. As one of the most important members of CFs, carbon nanofibers (CNFs) have been applied as promising materials in many fields, such as energy conversion and storage, reinforcement of composites and self-sensing devices [5–10].

**Figure 1.**
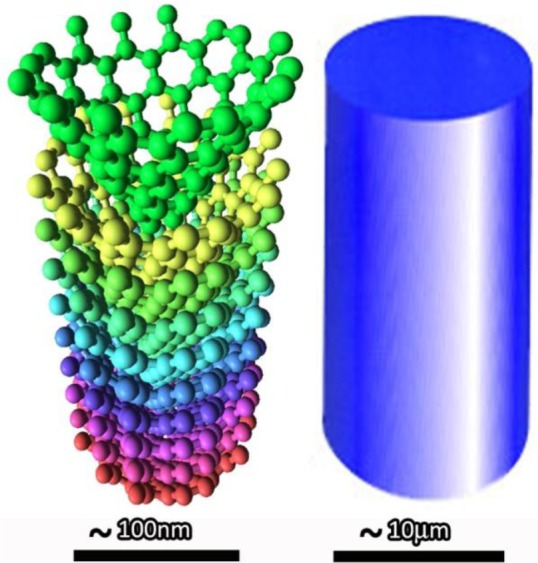
Schematic illustration of the difference between CNF and CCF.

There are some differences between conventional carbon fibers (CCFs) and CNF. The first one, also the most obvious one, is their size. The conventional CF has diameters of several micrometers, while CNFs have diameters of 50–200 nm. Figure 1 gives a schematic illustration of the difference between CNF and conventional CF. Except the diameter; the structures of the CNFs are evidently different from traditional carbon fibers. The typical CCFs were prepared from high-strength polyacrylonitrile (PAN) or meso-phase pitch (MP), varying the preparing conditions, including the oxidation atmosphere, the raw materials chosen and the heat treatment temperatures. The different preparing conditions will result in different properties of the prepared conventional CF. However, unlike the CCF, the CNF can be mainly prepared by two approaches: catalytically vapor deposition growth and electrospinning.

## 2. Synthesizing of CNFs

This section will discuss the preparation methods of the CNF. Currently, the CNF can be prepared mainly by two methods. One is catalytic thermal chemical vapor deposition growth, and the other one is electrospinning followed by heat treatment.

### 2.1. CNF Prepared by Catalytic Thermal Chemical Vapor Deposition Growth

**Figure 2.**
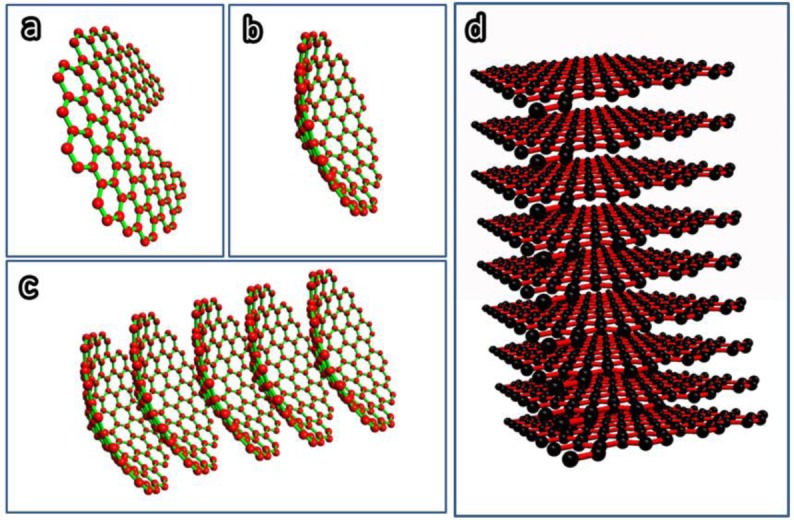
Schematic demonstration of (**a**–**c**) formation of cup-stacked CNF structure; and (**d**) platelet CNF structure.

Two types of CNF can be prepared by catalytic thermal chemical vapor deposition, namely, the cup-stacked CNF and the platelet CNF. The cup-stacked CNF, also called conical CNF, was first found by Ge and Sattler in 1994 [11]. Figure 2a–c gives the schematic demonstration of the formation of the cup-stacked CNF, and Figure 2d gives the schematic illustration of the platelet CNF structures.

**Figure 3.**
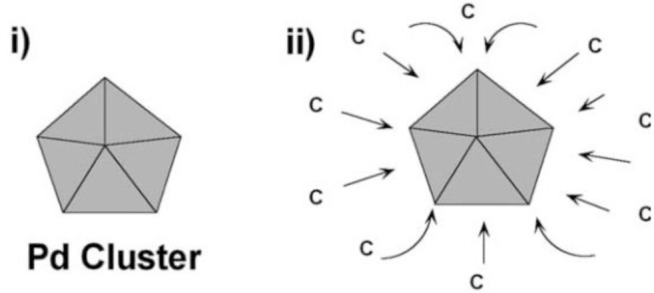

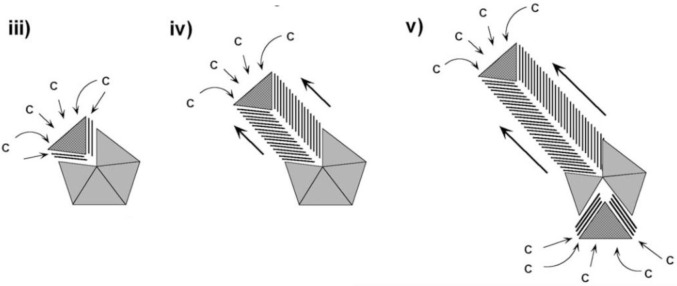
Schematic illustration of the typical chemical vapor deposition growth mechanism of the cup-stacked CNFs [14].

**Figure 4.**
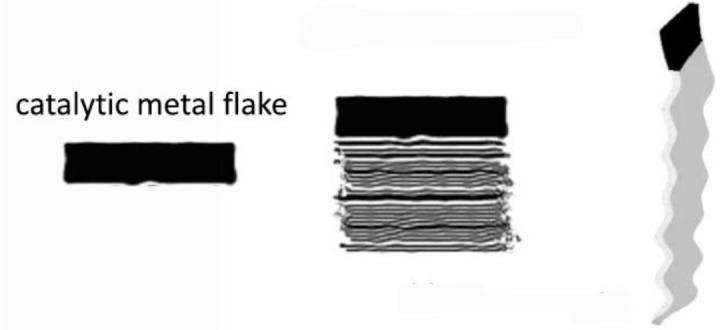
Schematic illustration of the typical chemical vapor deposition growth mechanism of the platelet CNFs [15].

**Figure 5.**
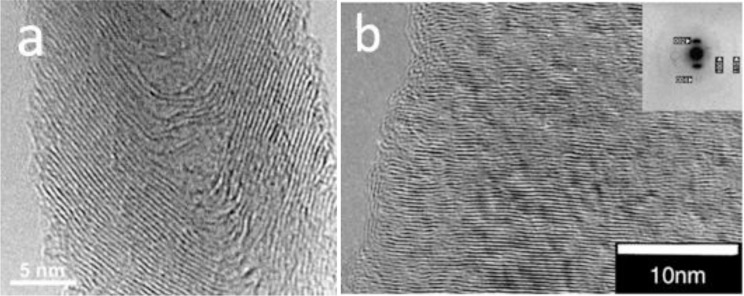
High resolution transmission electron microscope (HRTEM) image of (**a**) cup-stacked CNF and (**b**) platelet CNF [14,15].

For the preparation of CNF by the catalytic vapor deposition growth approach, several types of metal or alloys, which are able to dissolve carbon to form metal carbide, have been used as the catalyst, including iron, cobalt and nickel; chromium, and vanadium. Additionally, the molybdenum, methane, carbon monoxide, synthesis gas (H_2_/CO), ethyne or ethene are used to provide the carbon sources in the temperature range from 700 to 1200 K [12]. Generally, the structures of the CNF are governed by the shapes of the catalytic nanosized metal particles. The growth mechanism has been proven as the deposition of the hydrocarbons dissolved in the metal particle and precipitated on the metal surface as graphitic carbon [13]. Figures 3 and 4 demonstrate the schematic illustration of the typical growth mechanism of the cup-stacked and platelet CNFs [14,15]. Figure 5 demonstrates the high resolution transmission electron microscope (HRTEM) image of the cup-stacked CNF and the platelet CNF [14,15].

### 2.2. CNF Prepared by Electrospinning

Electrospinning is another widely used method for the preparation of the CNFs. Inagaki [16] recently reviewed CNF prepared by electrospinning process systematically. In this review, the CNFs prepared via electrospinning and carbonization was summarized according to their structure and properties. Most recently, Zhang [17] summarized the preparation and applications of CNFs prepared by electrospinning.

**Figure 6.**
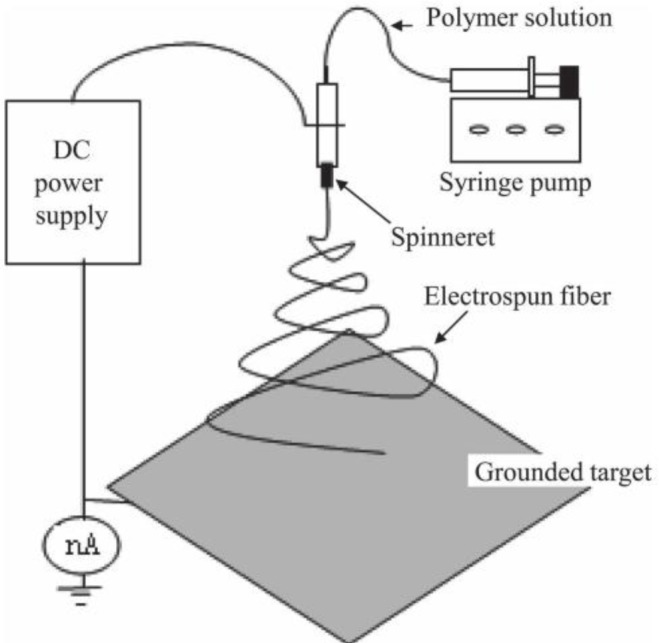
Schematic demonstration of the electrospinning setup for CNF fabrication [16].

To fabricate the CNFs by the electrospinning method, the polymer nanofibers are required to be prepared as the precursors of the CNFs. The properties of the final CNFs are decided by the types of polymer solution and the processing parameters. PAN and pitches are the most frequently used polymers. In addition, poly(vinyl alcohol) (PVA), polyimides (PIs), polybenzimidazole (PBI), poly(vinylidene fluoride) (PVDF), phenolic resin and lignin were also used [16]. Once the polymer nanofibers have been successfully prepared, a heat treatment will be applied to carbonize the polymer nanofibers to form CNFs. The morphology, purity, crystallinity, diameters and porosity are governed by the parameters of the heat treatment process, such as atmosphere and temperature. Figure 6 shows the schematic demonstration of the electrospinning device for CNF preparation.

After the polymer nanofibers have been successfully fabricated, the carbonization process will be followed by heating the polymer nanofiber up to 1000 °C in a specific environment. Generally, volume and weight change will occur during the carbonization process, which results in the decrease of the diameter of the CNFs.

In most cases, the CNFs prepared by the electrospinning method are prone to form web or mat structures. This structure is a good form to be used as electrode materials for batteries or supercapacitors. Due to the overall performances of the batteries largely counting on the transport performances of the ions in the electrolyte, therefore, controlling the pore structure is the most import factor to enhance the performances of batteries.

## 3. Preparation of CNF Composites

The overall performances of the CNF/polymer composites are largely governed by the dispersion of the CNF in the polymer matrix. Therefore, the dispersion technique plays a key role in the synthesizing of CNF composites.

The dispersion of CNF in polymer matrix can be realized mainly by two approaches: the melt mixing process and the sonication process in low viscosity solutions.

The most widely used method is melt mixing, due to its low cost, simplicity and availability. Generally, extrusion or roll mill [18,19], Haake torque rheometer [20] and mini-max molder [21,22] all belong to the melt mixing method. In this method, to obtain a good dispersion condition for CNF in polymer matrix, a high shear mixing condition is usually required. Although the high shear mixing will lead to a relatively good dispersion of the CNF, the aspect ratio, which is another key parameter governing the overall performances of the CNF/polymer composites, will be decreased during the mixing process. It was found that the decrease of the aspect ratio will result in the degradation of some properties [23,24]. Therefore, the investigation of the relatively low shear mixing approach without sacrificing the dispersion is still a challenge for the preparation of CNF/polymer composites by the melt mixing approach.

The chemical surface treatment of CNFs is a promising method to help their dispersion in the polymer matrix. In this process, the compatibility between the grafting functional groups and the polymer matrix is the key factor that decides the CNF dispersion and the overall performances of the composites. In most cases, the treatment process is oxidizing the CNF surface by soaking in sulfuric/nitric acid at various temperatures followed by acylation. After this process, the functional group will be grafted onto the surface of the CNF by the reaction between the oxidized CNF and the functional groups.

Li [25] and co-workers prepared and characterized the surface-treated CNF by using diamines or triamines as linker molecules. The amine group acts as a bridge connecting the CNF and the –NH_2_ to form the CNF–C(O)–NH– structure. Kelarakis and co-workers [26] prepared the CNF/ethylene/propylene (EP) random copolymer composite with a similar process. The as-received CNFs were surface oxidized by sulfuric/nitric acid and then reduced by sodium borohydride in absolute ethanol to form the CNF–OH structure. After that, the hydroxylated CNF was dispersed in dichlorobenzene and a polypropylene-graft-maleic anhydride polymer to form the CNF–O– structure.

Apart from the melt mixing processing for the CNF/thermoplastic polymers, the dispersion of the CNF in thermosetting polymers to prepare the CNF/thermosetting polymers (mostly epoxy resins) composites mainly counts on the solution approach with the help of sonication. In this process, the CNFs will be dispersed in the liquid epoxy form by sonication before being mixed with the hardener. Usually, acetone or other solutions are needed to help the effect of sonication. In addition, to avoid the increasing temperature during the sonication process, external cooling devices are necessary in most cases.

Pervin and co-workers [27] fabricated nanocomposites composed of SC-15 epoxy and CNF. The mixing process was carried out through a high-intensity ultra-sonication of the CNF and SC-15 epoxy. Once the sonication was completed, the hardener was added in the mixture, followed by high-speed mechanical stirring, and then cured at room temperature. The preparation of the CNF/epoxy nanocomposite by Choi *et al.* [28] showed that ozone surface treatment of the CNF is helpful for its dispersion in the epoxy matrix. In this study, CNF with and without ozone surface treatment was to investigating the dispersion conditions. The CNFs were dispersed in acetone by sonication and stirring process at room temperature. The epoxy resin was added into the CNF-acetone solution without stopping sonication and stirring. After this process, the acetone was removed by heating the mixture at 100 °C for 24 h, followed by the addition of the hardener, and then cured at room temperature.

## 4. Properties of CNF Composites

### 4.1. Electrical Conductivity

One of the most important properties of CNF composites is their electrical conductivity. When the CNF composites are applied as electrical devices, sensors, electromagnetic shielding or electrodes for batteries or supercapacitors, the electrical conductivity is always the first priority need to be considered.

In CNF composites, when the electrical conductivity measured as a function of the filling content of CNF, a typical “S” shape curve appears, due to the critical percolation phenomenon [29]. It follows a power law behavior expressed as[30]:

$$\begin{array}{l} \sigma_{\text{real}}=\sigma_{\text{c}}{\left(f-f_{\text{c}}\right)}^{t}p>p_{\text{c}};\\ \sigma_{\text{real}}=\sigma_{\text{c}}{\left(f_{\text{c}}-f\right)}^{-s}p<p_{\text{c}} \end{array}$$ (1)

where σ_real_ is the real electrical conductivity of the CNF composites; σ_c_ is the electrical conductivity of the CNF; *f* is the filling content of the CNF; *f*_c_ is the percolation threshold, which is defined as the critical value of the CNF filling content forming continuous network; and *t* and *s* are the electrical conductivity critical exponents above and below the percolation threshold, respectively. In this equation, σ_real_ was experimentally measured, and the values of σ_c_ and *f*_c_ are constants. According to the numerical and experimental results, it was claimed that, in the bond and site percolation lattices,the values of the conductivity critical exponents were universal, such that *t* ≈ 1.3–1.4, *s* ≈ 0.5 (in two dimensions) and *t* ≈ 1.6–2.0, *s* ≈ 0.6 (in three dimensions), based on the renormalization group theory [29,30], and in practical applications, they were usually considered belonging to the same universality system, as well. Some experimental and numerical results, however, have indicated that the practical application problems and the simulated lattice percolation problems do not belong to same universality systems. Although the percolation phenomenon has been studied for decades, the non-universality of the critical conductivity exponents observed experimentally has remained difficult to explain.

The Kogut and Straley (KS) model is a milestone for quantitatively analysis of the critical conductivity exponents regarding the non-universality in a percolation system [31]. It first claimed that the universality of the conductivity exponents would be broken if the low-conductance bonds in percolation networks were characterized by an anomalous conductivity distribution. This model was derived from the mean field theory by assigning each neighboring pair in a regular lattice. It was claimed that, in a lattice percolation system, if a bond with finite conductivity, *g*, with probability μ and zero conductivity with probability 1 *−* μ, the bond conductivity distribution function can be written as:

ρ(g)=μh(g)+(1−μ)δ(g) (2)

where δ(*g*) is the Dirac delta function and *h*(*g*) is the distribution function of the finite bond conductivity. If *h*(*g*) has a power law divergence for small *g* of the form:

$$\underset{g\to 0}{\text{lim}}h\left(g\right)\propto g^{-\alpha }$$ (3)

where α ≦ 1, then the universality of the conductivity critical exponents will be lost with sufficiently large values of exponent α.

**Figure 7.**
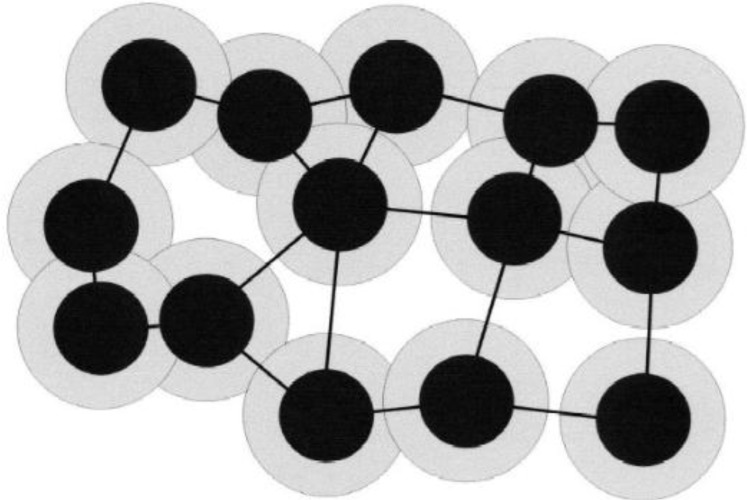
Schematic demonstration of the tunneling model in carbon/polymer composite [33].

Based on the KS model, a well-known model, namely the “tunneling model”, was introduced by Balberg [32], corresponding to granular materials and carbon/polymer composites. Figure 7 shows the schematic demonstration of the tunneling model, which is the main conductivity mechanism of the carbon/polymer composites. As demonstrated in this figure, the black spheres represent the carbon materials, and the grey circles around the black spheres represent the polymers. In this model, the electrical conductivity of the carbon/polymer composites is governed by the width of the tunnel, which means the thickness of the polymer layer on the surface of the carbon materials.

In CNF composites, the tunneling effect is the main mechanism of electrical conduction; therefore, the electrical conductivity of the CNF/polymer composites are affected by the thickness of the polymer layer on the CNF surfaces, which is decided by the surface treatment methods and the polymer types. Although the tunneling model has been widely used in describing the non-universality of the percolation system; the conductive fillers were assumed as spherical particles; thus, it is insufficient to describe the percolation system with non-spherical fillers, such as CNF-filled polymer composites.

In recent years, a comprehensive understanding of the mechanism of conduction in CNF/polymer composites was developed rapidly thanks to the development of random media physics [33–37]. The electrical conductivity was systematically investigated via theoretical models and the microstructure dependence of the carbon polymer composites. Lux [38], Kirkpatrik [39], Clerc [40] and Nan [29] reviewed the physics of percolation theory and the physics of inhomogeneous materials.

Another important model to describe the percolation phenomenon in a conductor-insulator composite is the general effective media (GEM) model, which was described by McLachlan [41] in detail. In the GEM model, the composite was considered as a symmetric medium, in which the conductor with an ellipsoidal or spherical shape was embedded in the insulator matrix. Under this condition, the relationship of the conductivities between the composite, the insulator matrix and the conductive fillers can be written as:

$$\frac{p(\sigma_{l}-\sigma_{m})}{\sigma_{l}+\left(\frac{p_{c}}{1-p_{c}}\right)\sigma_{m}}+\frac{\left(1-p)(\sigma_{h}-\sigma_{m}\right)}{\sigma_{h}+\left(\frac{p_{c}}{1-p_{c}}\right)\sigma_{m}}=0$$ (4)

where *p* is the conductor’s filling content; *p*_c_ is the percolation threshold; and σ_h_, σ_l_, and σ_m_ are the electrical conductivities of the conductor, insulator and the composite, respectively.

It has been widely accepted that, if the effective conductivities are given by the average conductance values, from the perspective of the geological structures, the conductivities can be divided into two types: the series and the parallel. In CNF composites, one extreme case is that all CNFs in the composite connected in parallel form, where the equivalent effective conductivity can be written as:

$$\sigma_{\text{eff}}\text{∞}{\left(\sum_{i}R_{i}\right)}^{\left(-1\right)}\text{∞}{\left(\sum_{i}\sigma_{i}^{\left(-1\right)}\right)}^{\left(-1\right)}$$ (5)

In this assumption, if all CNFs are geometrically identical, the σ_eff_ will be proportional to the second sum.

Another extreme case is that all CNFs are connected in series form, where the equivalent effective conductivity should be written as:

$$\sigma_{eff}\propto \sum_{i}\sigma_{i}$$ (6)

Of course, in a real CNF composite, the resistance of the whole system is not able to be reflected only by parallel or series form; therefore, the real connection form or conduction channel has to be analyzed. In addition, there is a geometrical restriction in this case, that the CNFs must all be congruent, which means all the CNFs have to be of the same size and shape; otherwise, the geometrical factors have to be included in the analysis [31].

In a CNF composite, the continuous network of the CNF can be categorized into two types: the backbone and dangling ends [42,43],which show different properties. The percolation backbone is to demonstrate the real path that carries the current transport. In previous studies, it was found that the effective path or minimum length of the conduction was governed by the backbone based on widely simulated results in different lattice percolation systems [43]. Although these results are able to reflect the backbone characteristics to some extent [44], they were still hardly able to analyze the real conductor-insulator composite, due to the backbone structure being very hard to be directly observed experimentally.

In a CNF/polymer composite, near the percolation threshold, not all CNFs belong to the continuous percolation backbone, because some of them still belong to the isolated clusters or form dangling ends. Therefore, the contribution to the composite properties of the percolation backbone density, which is defined as the proportion of the continuous percolation network in the whole percolation infinite clusters, is not the same as the isolated cluster.

We presented a new model to describe a possible non-universal behavior in a conductor-insulator composite system [45]. In this model, the backbone and dangling end masses, *M_B_* and *M_D_*, were presented as the key parameters to describe the backbone structure. The backbone or dangling end density is defined as the portion of the total backbone or dangling ends that belong to the percolation infinite clusters, respectively. In a percolation system, the conductance between two randomly selected nodes (*g*) can be expressed as:

$$g=g_{0}\exp\left(-\kappa \frac{M_{D}}{M_{B}}\right)$$ (7)

where *g*_0_ is a constant; *M*_D_ and *M*_B_ are dangling ends and backbone masses belonging to two randomly selected nodes; and κ is called the “structural factor” that represents the geometry and topology structure of the conductor. According to this assumption, the value of κ was defined as the function of the aspect ratio of the fillers and expressed as:

$$\kappa =\frac{1}{\sum^{\text{​}}f_{i}{\left(\log\left|{\left(\frac{a}{b}\right)}^{\lambda }\right|+1\right)}_{i}}$$ (8)

where *f* is the filling content of the conductive fillers; *i* is the index of the filler types; *a/b* represents the aspect ratio of the filler; and λ is 1, 0 and −1 correspond to *a* > *b*, *a* = *b* and *a* < *b*, respectively. In this equation, the tunneling conductance parts should be considered as a part of the backbone.

Our recent study demonstrates that the conductive distribution function of the percolation network, *H* (*M*_D_*/M*_B_), and the dangling ends and the backbone masses could be expressed with the exponential form [45]:

$$H\left(\frac{M_{D}}{M_{B}}\right)\propto \frac{\eta^{2}M_{D}}{M_{B}}\exp\left(-\frac{\eta M_{D}}{M_{B}}\right)$$ (9)

where η is the average value of $\frac{M_{\text{B}}}{M_{\text{D}}}$, written as:

$$\eta =\langle \frac{M_{B}}{M_{D}}\rangle$$ (10)

Therefore, the critical exponent, *t*, can be obtained as:

$$t=t_{\text{un}}+\kappa \langle \frac{M_{B}}{M_{D}}\rangle -1=t_{un}+\left[\frac{1}{\sum^{\text{​}}f_{i}{\left(\log\left|{\left(\frac{a}{b}\right)}^{\lambda }\right|+1\right)}_{i}}\right]\langle \frac{M_{D}}{M_{B}}\rangle -1$$ (11)

where *t*_un_ is the “universal value” of a percolation system based on the effective medium theory [31]. In this equation, if there is no backbone in the system, the *t* value goes to infinity, and the electrical conductivity goes to zero. In this extreme case, the system is an insulator. If there are no dangling ends in the system, the *t* value goes to zero, which means that the electrical conductivity goes to σ_c_. In this extreme case, the system is a pure conductor. According to this model, the effective electrical conductivity of the system increases with the decreasing of the conductivity critical exponent, which is a key factor to reflect the backbone density of the fillers.

**Figure 8.**
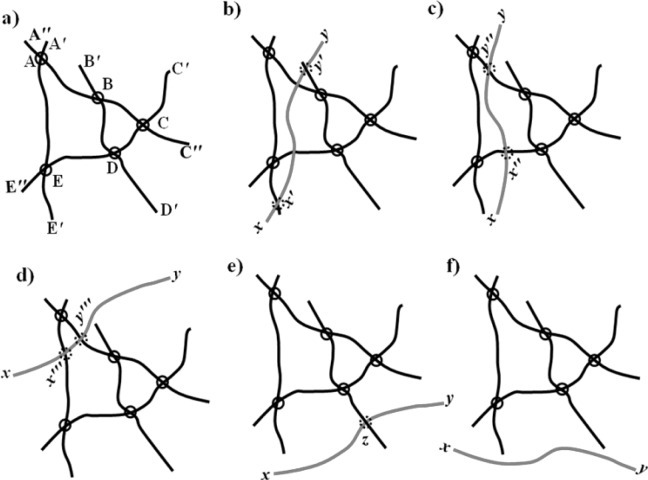
Schematic illustration of the backbone structure variation mechanisms in the CNF infinite cluster with the addition of CNF above the percolation threshold: (**a**) original infinite cluster; (**b**) the increased CNF locates on the loop and the connected part of the previous dangling ends; (**c**, **d**) the increased CNF locates on the loop, but does not connect any previous dangling ends; (**e**) the increased CNF belongs to the infinite cluster, but neither locates on the loop nor connects any previous dangling ends; (**f**) the increased CNF is isolated and does not belong to the infinite cluster [46].

Based on this analysis, the backbone variation trend with increasing filling content of the CNF was analyzed [46]. Figure 8 gave the schematic illustration of the backbone structure variation mechanisms with the increasing content of the fillers with the long aspect ratio above the percolation threshold.

This figure shows the original infinite cluster of the filler with long aspect ratio in a composite. As can be seen in the figure, the loop, *ABCDE*, belongs to the backbone, *M_B_*, and *AA′*, *AA″*, *BB*′, *CC*′, *CC*″, *DD*′, *EE*′ and *EE″* belong to the dangling ends, *M_D_*, in the infinite cluster. As such, the ratio of the backbone and the dangling ends is:

$$\frac{M_{B}}{M_{D}}=\frac{AB+BC+CD+DE+EA}{AA^{\prime }+AA^{\prime \prime }+BB^{\prime }+CC^{\prime }+CC^{\prime \prime }+DD^{\prime }+EE^{\prime }+EE^{\prime \prime }}$$ (12)

Figure 8b–f categorized the backbone structure variations with increasing filling content based on Figure 8a. As shown in Figure 8b, the added part, *xy*, was located on the percolation loop and connected the previous dangling ends, *BB*′ and *EE*′. As a result, the mass ratio between the backbone and the dangling ends changed to:

$$\frac{M_{B}}{M_{D}}=\frac{AB+BC+CD+DE+EA+xy}{AA^{\prime }+AA^{\prime \prime }+CC^{\prime }+CC^{\prime \prime }+DD^{\prime }+EE^{\prime \prime }+xx^{\prime }+yy^{\prime }+B^{\prime }y^{\prime }+E^{\prime }x^{\prime }}$$ (13)

In Figure 8 c,d, the added part was located on the loop, but did not connect any previous dangling ends; therefore, the backbone density changed to:

$$\frac{M_{B}}{M_{D}}=\frac{AB+BC+CD+DE+EA+x^{\prime \prime }y^{\prime \prime }}{AA^{\prime }+AA^{\prime \prime }+BB^{\prime }+CC^{\prime }+CC^{\prime \prime }+DD^{\prime }+EE^{\prime }+EE^{\prime \prime }+xx^{\prime \prime }+yy^{\prime \prime }}$$ (14)

under this condition, the variation of the backbone density depended on the length ratio of *xx″* +*yy″*, *x*″*y*″ in Figure 8c and *xx*′″ + *yy*′″ and *x*′″*y*′″ in Figure 8d.

In Figure 8e, the added part belongs to the infinite cluster, but was neither located on the loop nor connected to any previous dangling ends. In Figure 8f, the newly added *xy* was isolated and did not belong to the infinite cluster. Therefore, the backbone density changed to:

$$\frac{M_{B}}{M_{D}}=\frac{AB+BC+CD+DE+EA}{AA^{\prime }+AA^{\prime \prime }+BB^{\prime }+CC^{\prime }+CC^{\prime \prime }+DD^{\prime }+EE^{\prime }+EE^{\prime \prime }+xy}$$ (15)

In this condition, the backbone density decreased with the increase in CF concentration.

Fractals, as another important tool, have been widely applied to quantitatively describe the composites of randomly distributed fillers with complex and irregular geometries embedded in a matrix [47]. The fractal characterization of the fillers can be divided into regular and irregular types. The regular fractal is self-similar in all scale ranges, while the irregular fractal is only self-similar in a certain scale range. The typical irregular fractal is a heterogeneous object, such as the CNF in a polymer matrix. In this system, near the percolation threshold, the geometry of the CNF is able to be reflected by an irregular fractal.

**Figure 9.**
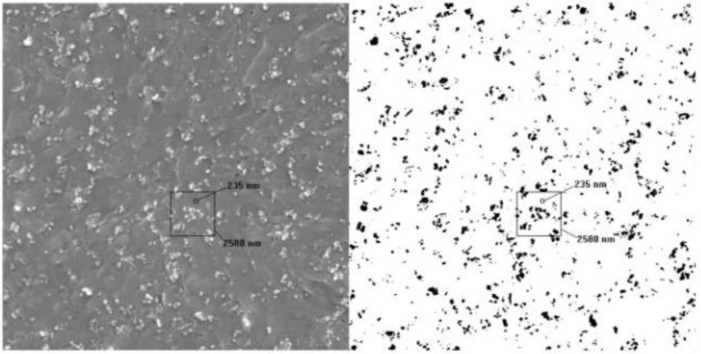
SEM image and its binarized image for the fractal dimension calculation in the carbon/polymer composite [52].

**Figure 10.**
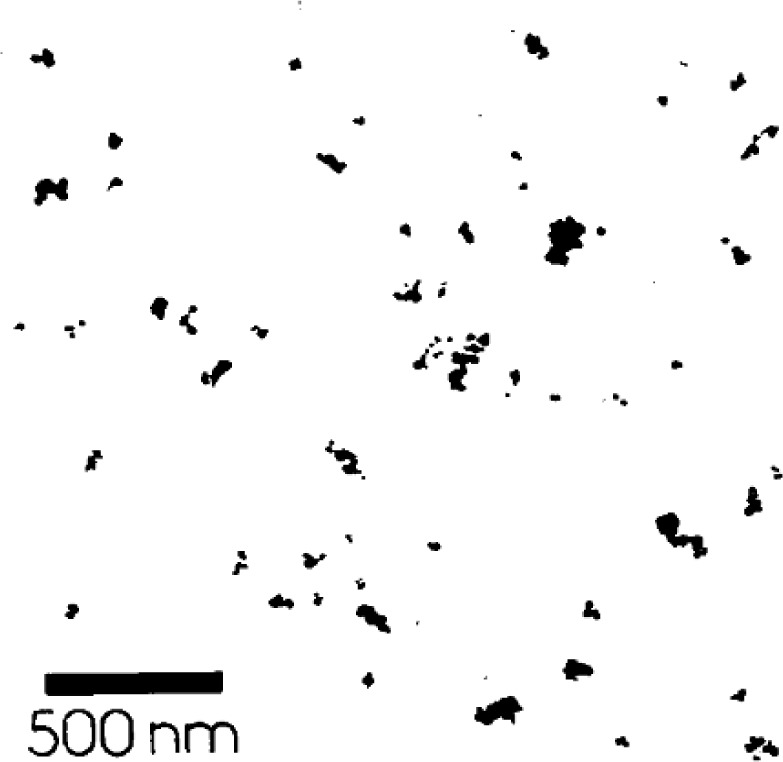
TEM image for the fractal dimension calculation in the carbon/polymer composite [53].

Numerical works have demonstrated that the infinite clusters with fractal dimensions of *D* ≈ 1.9 and 2.5 [48] are able to be determined, and the optimal path with fractal dimensions of *D* ≈ 1.22 and 1.43, corresponding to 2D and 3D lattices, are also able to be detected in the infinite clusters, respectively [49]. In a real composite, microscopy images, including transmission electron microscopy (TEM), scanning electron microscopy (SEM) and optical microscope (OM), are all able to be used to determine the fractal dimension of the fillers directly [50,51].Figures 9 and 10 show the SEM and TEM images of carbon/polymer composites, which were used to calculate the fractal dimensions of the carbon fillers in the polymer matrix [52,53].

In addition, the same as the infinite cluster of the fillers, the geometry of the backbone cluster also can be reflected by fractals. The investigation of the backbone structure of the CNF is very important for understanding the transport properties of the CNF composites. An effective method, called the “burning method”, was developed to extract the elastic backbone from the infinite clusters in a percolation system [54]. The fractal dimension of the elastic backbone quantitatively describes the structure and irregularities of the effective continuous network and paths that are accounting for the conduction in a disordered media.

In CNF/polymer composites, there are several topics that were investigated in a limited manner. To the best of our knowledge, the quantitative analysis of the CNF distribution and connection in CNF composites based on fractal analysis is still at beginning stages; in addition, little discussion has focused on the non-universality of the conductivity critical exponents. A lot of work needs to be developed in future studies to investigate the CNF distribution and conductivity critical exponents that helps to enhance the electrical properties of the CNF composites.

### 4.2. Thermal Conductivity

Similar to the electrical conductivity, the thermal conductivity is another important property of the CNF composite, and the thermal transport phenomenon has been widely studied, as well, especially the thermal conductivity as a function of the filling content of the CNFs.

To increase the thermal conductivities of the phase change materials, CNF and CNT were mixed in soy wax and paraffin wax with dosages of 1, 2, 5 and 10 wt% to prepare the phase change composites [55]. It was found that the thermal conductivities of the phase change materials increased from 0.324 to 0.469 W/mK with the CNF filling content increasing from 0 to 10 wt%.

Teng [56] tested the thermal conductivities of the CNF/polylactic acid (PLA) and CNT/PLA-PLA composites that were prepared with a Haake torque rheometer equipped with an electrically heated mixing head and two non-interchangeable rotors. In these composites, the CNFs are randomly distributed in the PLA matrix, and it was found that the thermal conductivity of the composite reached 1.2 W/mK with a CNF filling content of 10 wt%, which is much higher than the neat PLA.

Other than the randomly distributed CNF composites, CNF paper-like mats were prepared by Mahanta and Abramson [57]. Unlike the randomly distributed CNF composites, the thermal conductivities of these CNF mats exhibit a high in-plane direction and a low through-plane direction. The in-plane thermal conductivity varied from 12 to 157 W/mK with a filling content of 6.7% and 46.2%, respectively, while the through-plane thermal conductivities were measured as 0.428 and 0.711 W/mK.

A lot of models were developed to predict the thermal conductivity of the composite. The Maxwell model [58] is one of the most widely applied ones, which claimed the thermal conductivity of a composite could be predicted as:

$$\rho_{\text{mix}}=\rho_{\text{const}}\frac{1+2\theta -2f(\theta -1)}{1+2\theta +f(\theta -1)}$$ (16)

where ρ_mix_ is the thermal conductivity of the composite; θ is the ratio of the thermal conductivity of the matrix and the fillers; and ρ_const_ and *f* are the thermal conductivity and the volume fraction of the fillers, respectively. Another famous description is the series/parallel model [59]:

$$\rho_{\text{mix}\left(p\right)}=\rho_{d}f_{d}+\rho_{c}f_{c}$$ (17)

$$\rho_{\text{mix}\left(s\right)}=\frac{\rho_{d}\rho_{c}}{\rho_{d}f_{c}+\rho_{c}f_{d}}$$ (18)

where ρ_mix_ is the thermal conductivity of the composite, ρ_c_ and ρ_d_ are the thermal conductivities of the matrix and the filler and *f*_c_ and *f*_d_ are the filling content of the matrix and the fillers, respectively. Recently, based on experimental analysis, an analytical model was developed to predict the thermal conductivity of a carbon nanotube-graphene/epoxy composite. This model built a bridge to connect the hybrid structure of the fillers to the thermal conductivity of the composite [60,61]. In this model, the thermal conductivities of the both CNT and the graphene were considered as parameters, and the thermal conductivity of the final CNT/GR/epoxy hybrid composites can be predicted as:

$$\rho_{\text{mix}}=\frac{1+f_{c}\left(\frac{\rho_{c}}{\rho_{e}}\right)/3+2{\left(f_{g}-0.001\right)}^{a}\left(\frac{\rho_{g}}{\rho_{e}}\right)/3}{1-(2f_{c}+f_{g})/3}$$ (19)

where ρ_mix_, ρ_c_, ρ_g_ and ρ_e_ represent the thermal conductivities of the composite, carbon nanotubes, graphene and epoxy, respectively, and the *f*_c_ and *f*_g_ represent the volume fractions of the carbon nanotubes and the graphene.

These models have successfully built a bridge to connect the thermal conductivity and the content of the fillers in composites; however, the effects of the geometrical tortuosity, which reflects the real transport channels in a CNF composite, on the thermal conductivity still require systematic studies, and the prediction of the thermal conductivity of the CNF composites as a function of the geometrical structure of the CNF is still one of the most challenging topics in CNF composites.

It is well known that the electrical transport properties obey the power law near the percolation threshold [45], and the geometrical structure of the fillers shows self-similarity in a scale range and is able to be characterized by fractal dimensions [47]. Numerical works demonstrated that the fractal dimensions of the infinite clusters are *D* ≈ 1.9 and 2.5, and the fractal dimension of the shortest path in the infinite clusters are *D* ≈ 1.22 and 1.43, corresponding to 2D and 3D dimensional lattices, respectively [62].

Unlike the electrical conductivity, the thermal conductivity as a function of the filling content is not able to be reflected by only applying the power law and the fractal dimension, due to the thermal conductivity percolation phenomenon not being evident in the system near the percolation threshold [63]. Therefore, effective models have to be developed to quantitatively determine the thermal conductivity as a function of the filling content of the fillers. Moreover, the microstructure characterization via fractal geometry has to be examined carefully, since the final properties largely count on the structure of the matrix and the fillers in the composite.

Recently, Coleman [64] presented a model that connected the tortuosity, fractal and filling content of the fillers. In this work, it was claimed that the tortuosity obeys the power law with a form of τ ~ *f*^−^^ε^, and the exponent, ε, needs to be accurately predicted by new fractal characteristics. In addition, although the difference of the topological dimension and the fractal dimension is able to reflect the geometrical tortuosity of the conductor, the thermal conductivity is not available to be quantitatively determined only by the fractal dimension as a result of being increased with the increasing filling content of the conductor phase. Therefore, the same fractal dimension might appear in two different composites with different filling contents and different geometrical structures.

### 4.3. Mechanical Properties

The mechanical properties of the polymer reinforced by adding carbon nanomaterials have been investigated for decades [65]. Generally, the mechanical properties of the polymer will increase with increasing CNF content. Bortz [66] reported that the resistance to fracture increased by 66% and 78%, with a 0.5 wt% and 1.0 wt% addition of CNFs in CNF/epoxy composites. The CNF-reinforced thermal-plastic polyurethane (TPU) composites were prepared by the melt mixing method [67]. In this composite, the tensile strength of the nanocomposites increased 49% with the CNF filling content of 4 wt% compared with the neat TPU. However, the tensile strength of the CNF-reinforced nanocomposites reduced dramatically with a higher CNF dosage, because of the increase in voids or other defect densities resulting from the CNF bundles, which will lead to stress concentration under dynamic loading and finally, results in a premature failure at low strain values.

Apart from the strengths or modulus, the microhardness of the CNF composites was also found to increase with increasing CNF content [68]. The microhardness of the epoxy matrix improved by 53% with 0.5 wt%, 62% with 0.75 wt% and 100% with 1.0 wt% addition of CNFs. The high aspect ratio and high modulus strength of CNFs contributed to the reinforcement, thus improving the hardness value.

The reinforcement mechanisms have been systematically studied, as well, and many successful results have been extensively reported to demonstrate the relationship between the mechanical properties of the composites and the reinforcement fillers. The most widely accepted model is the mixture model expressed as:

$$\rho_{\text{mix}}=\rho_{r}f_{r}+\rho_{m}f_{m}$$ (20)

where ρ_mix_ is the mechanical properties of a composite, such as strength, Young’s modulus or fracture toughness, *f*_r_ and *f*_m_, and ρ_r_ and ρ_m_ are the filling contents and the mechanical properties of the reinforcement phase and the matrix, respectively. Especially, if the reinforcement materials are fibrous materials, such as CNTs or CNFs, this model should be modified as [69]:

$$\rho_{\text{mix}\left(p\right)}=\mu_{1}\mu_{2}\rho_{r}f_{r}+\rho_{m}f_{m}$$ (21)

where μ_1_ represents the length efficiency factor and μ_2_ represents the orientation efficiency factor.

The Cox model is a typical model to predict the Young’s modulus of CNF-reinforced polymer based on the rule of mixtures [70]. It was written as:

$$E_{c}=(1-V_{f})E_{m}+nV_{f}(\rho_{f}E_{f})$$ (22)

$$\rho_{f}=1-\frac{\tan h\beta }{\beta }$$ (23)

$$\beta =\frac{l}{d}\sqrt{\frac{E_{m}}{(1+\nu )E_{f}\ln\sqrt{\left(\frac{\pi }{4V_{f}}\right)}}}$$ (24)

where *E*_c_ is the modulus of the composite; *V*_f_ is the filling content of the filler; *E*_m_ is the Young’s modulus of the polymer matrix; ρ_f_ is the efficiency factor of the filler; *E*_f_ is the Young’s modulus of the filler; *n* is an orientation constant (*n* = 1/6 for random orientated fibers in 3D, *n* = 1/2 for random orientated fibers in 2D and *n* = 1 for aligned fibers); *l* is the average fiber length; *d* is the diameter and ν is Poisson’s ratio.

Other models based on the rule of mixtures were further developed in fibrous, reinforced composites. The elastic modulus of the composites can be expressed as [71]:

$$E_{c/m}=\left(E_{r/m}-1\right)V_{r}(\text{lower bound})$$ (25)

$$E_{c/m}=\frac{\left(E_{r/m}-1\right)V_{r}}{E_{r/m}+(1-E_{r/m})V_{r}}(\text{upper bound})$$ (26)

where *V_r_* is the filling content of the reinforcement; *E*_c/m_ = *(E*_c_ *− E*_m_*)/E*_m_; *E*_r/m_ = *E*_r_*/E*_m_; and *E*_c_; *E*_r_; and *E*_m_ are the longitudinal elastic moduli of the composites, reinforcement phase and the matrix, respectively. However, some experimental results claimed that the mechanical property of composites as a function of reinforcement filling content is not linear, especially when the filling content takes large values. Consequently, a revised form of the rule of mixtures was developed with an exponential form [71]:

$$E_{c/m}=\left(\varepsilon_{l}\varepsilon_{o}{\text{ε}}_{w}E_{\frac{r}{m}}-1\right)V_{r}\exp\alpha V_{r}$$ (27)

where:

$$\alpha =\frac{\text{lnβ}}{{\hat{V}}_{r}},\;\beta =\frac{{\hat{E}}_{c/m}}{\left({\hat{\varepsilon }}_{l}\varepsilon_{0}\varepsilon_{w}E_{r/m}-1\right){\hat{V}}_{r}},\;{\hat{E}}_{c/m}=({\hat{E}}_{c}-E_{m})/E_{m}$$ (28)

The hat sign means that the values need to be determined by experimental results, and ε*_l_*, ε_0_ and ε_w_ are constants.

Another well-accepted model is the Halpin–Tsai equation [72–74], which gives the prediction of the tensile modulus of nanocomposites, expressed as:

$$\begin{array}{l} E_{c}=\left[\frac{3}{8}\times \frac{1+2\left(\frac{l}{d}\right)\rho_{L}V_{r}}{1–\rho_{L}V_{r}}+\frac{5}{8}\times \frac{1+2\rho_{D}V_{r}}{1–\rho_{D}V_{r}}\right]E_{m},\\ \rho_{L}=\frac{\left(\frac{E_{r}}{E_{m}}\right)-\left(\frac{d_{m}}{4t}\right)}{\left(\frac{E_{r}}{E_{m}}\right)+\left(\frac{l_{m}}{2t}\right)},\;\rho_{D}=\frac{\left(\frac{E_{r}}{E_{m}}\right)–\left(\frac{d_{m}}{4t}\right)}{\left(\frac{E_{r}}{E_{m}}\right)+\left(\frac{d_{m}}{2t}\right)} \end{array}$$ (29)

Where *E*_c_ and *E*_m_ represent the Young’s moduli of the composite and the matrix; *l/d* and *V*_m_ are the aspect ratio and the filling content of the nanofibers or nanotubes; and *t* is the thickness of the graphite layer.

Apart from the models mentioned above, the “composite sphere method” (CSM) was also developed to predict the modulus of composites, which can be written as [75]:

$$K_{\text{com}}=K_{r}+\frac{V_{m}}{\frac{1}{K_{m}-K_{r}}+\frac{3V_{r}}{\left(3K_{r}+4G_{r}\right)}}$$ (30)

$$G_{\text{com}}=G_{r}+\frac{V_{m}}{\frac{1}{K_{m}-K_{r}}+\frac{6V_{r}}{\left(3K_{r}+2G_{r})/(5G_{m}(3K_{r}+4G_{r})\right)}}$$ (31)

where *K*_com_ and *G*_com_ are the bulk and shear moduli of composites; *V*_m_ and *V*_r_ are the filling content of the matrix and the reinforcement phase; *K*_m_, *G*_m_ and *K*_r_, *G*_r_ are the bulk moduli and shear moduli of the matrix and the reinforcement phase, respectively.

Although the mechanical properties of CNF reinforced composites were extensively studied, and numerous methods were applied to characterize the relationship between the fillers and the final properties, the disagreement between some experimental results and theoretical models, which resulted from the complexity of the reinforcement phase, is always a problem that needs to be solved, especially that of how to quantitatively predict the mechanical properties of the composites with different geometrical structures of reinforcements, which is still one of the most challenging topics in composite science and technology. Further studies need to be focused on the influence of the geometrical structure of the CNF on the mechanical properties of the composites.

One thing to be noted here is that the overall properties of CNF-modified composites are largely decided by the CNFs-matrix interfaces. Cu/CNF composites with high thermal conductivity were prepared via three different powder metallurgy approaches [76]. It was found that the interfaces are very clear and strong, and a high density of dislocations of the Cu matrix can be observed at the interface area, which is an important factor that decides the thermal properties of the composites. Lu [77] prepared CNF/epoxy-based shape memory polymer composites, and the electrical and thermal properties of the composites were tested. It was also claimed that the CNF/polymer interfaces play the key role in the final properties of the composites. Sandler [19] studied the mechanical properties of vapor growth CNF-reinforced poly-ether-ether-ketone (PEEK) composites. The interfaces between the CNFs and the PEEK were observed by SEM and TEM. It was found that the interface remained intact during the preparing process, which guarantees the strengthening effect of the CNFs.

In spite of many experimental studies having demonstrated that the interface between the CNFs and the matrix is considerably important to the overall performances, limited theoretical models have been developed to quantitatively explain the effect of the interfaces on the properties, such as the bonding types, bonding strengths and dislocation densities. The bonding between the CNF and the matrix is different from the CCF and CNT due to the different external surface structures of CNFs, CCFs and CNTs (shown in Figure 1). This should be considered as an important factor before developing a theoretical model to explain the relationship between the performances and the CNF/matrix interfaces.

## 5. Applications

### 5.1. Sensors

Generally, the self-sensing function of the CNF composites is realized by testing the variation of electrical properties that has resulted from the change of the external conditions, including stress/strain and the gas environment. The electrical conductivity of the CNF composites is able to be reversibly changed by several orders of magnitude with the reversible change of the external conditions.

Zhu [78] prepared CNF/elastomer (VM2) composites with a percolation threshold of 1 wt% as strain sensors for reflecting large mechanical deformation. The electrical conductivity of the composite is able to reversibly change 10^2^–10^3^ orders of magnitude upon stretching to 120% strain and recovery to 40% strain.

**Figure 11.**
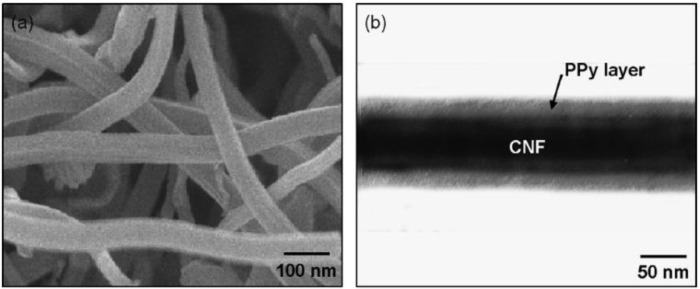
SEM and TEM images of the PPy-coated CNF as a toxic gas sensor [80].

CNF/poly(acrylate) was prepared by Li [79] as gas sensors. In this study, the vapor can be detected via a five orders of magnitude change of the electrical conductivity. Other than vapor sensors, the CNF/(polypyrrole) PPy coaxial nanocable toxic gas sensor was fabricated to perceive irritant gas, such as NH_3_ and HCl, via the one-step vapor deposition polymerization method [80]. The structure of the sensor is composed of an ultrathin and uniform PPy layer on the surface of CNF, shown in Figure 11. By the change of the oxidation level of the PPy layer resulting in the reaction between the PPy and the NH_3_ or HCl, the electrical conductivity of the composite decreased because of the decrement in the charge carrier density.

Apart from the strain/stress or gas sensing, the temperature, humidity, magnetic field and light are all important factors that are required to be sensed in many applications. The CNF and their composites for sensing these factors still need to be investigated deeply in future studies.

### 5.2. Batteries

Currently, the CNF composites as electrode materials for batteries and supercapacitors have been widely studied worldwide. The main requirement for high performance batteries and supercapacitors is high porous electrode materials, which are able to contain enough electrolytes and satisfy the fast and long-term ion transport.

**Figure 12.**
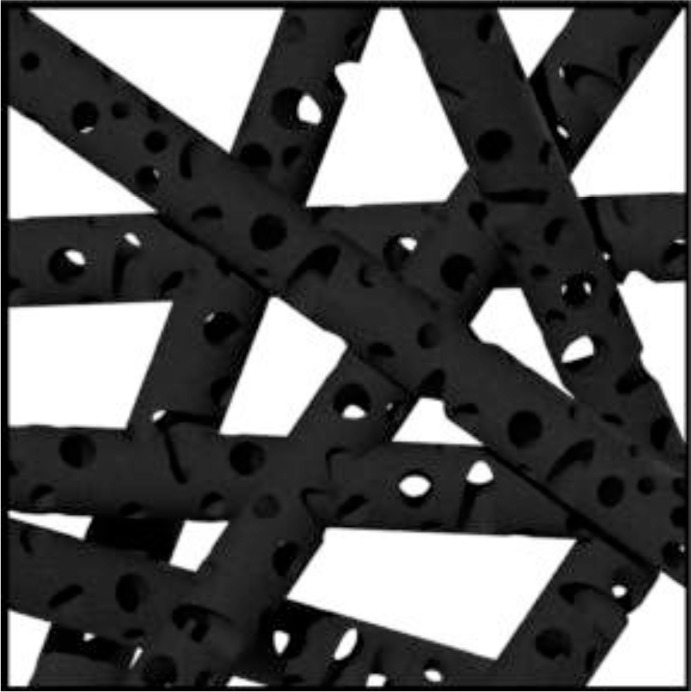
Schematic demonstration of the porous CNF prepared by the carbonization of electrospun PAN/SiO_2_ composite nanofibers followed by resolving the SiO_2_ nanoparticles with HF [81].

Ji [81] prepared a porous CNF by the carbonization of electrospun PAN/SiO_2_ composite nanofibers followed by resolving the SiO_2_ nanoparticles with hydrofluoric acid (HF). This porous CNF has magnified surface areas and defects. The specific porous structure guarantees that this CNF is able to be used as an anode material for lithium ion batteries directly, without adding any polymer binder or non-active carbon black. Figure 12 shows the schematic illustration of the porous CNFs.

**Figure 13.**
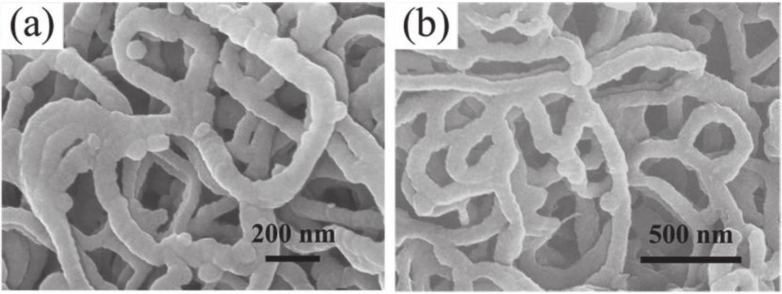
The SEM microstructure of the (**a**) PPy nanofiber webs and (**b**) CNF webs [82].

A facile way to synthesize N-dope porous CNF webs as anode materials for lithium ion batteries via using polypyrrole as a precursor was reported by Qie [82]. The high-level N-doping and the nanostructure of the CNF webs guaranteed a reversible capacity of 943 mAh/g with a current density of 2 A/g, even after 600 cycles. Figure 13 gives the SEM microstructure of the (1) PPy nanofiber webs and (2) CNF webs.

**Figure 14.**
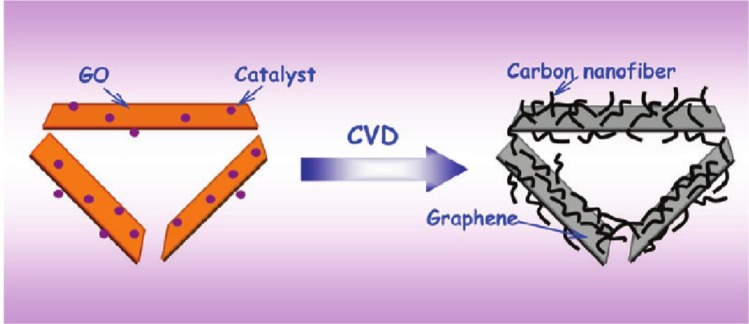
Schematic illustration of the structure with 1D CNF grown on 2D graphene nanosheets [83].

A 3D CNF/graphene nanosheets hybrid material was prepared via the chemical vapor decomposition approach by Fan [83]. This is a special structure with the 1D CNF grown on the 2D graphene nanosheets that contains sufficient cavities, open tips and exposed edges of the graphene sheet. Figure 14 gives the schematic illustration of the structure with the 1D CNF grown on the 2D graphene nanosheets. Due to this 1D–2D hybrid structure, the lithium ions could be stored in these spaces more efficiently, which guaranteed the high reversible capacity (667 mAh/g), high-rate performance and cycling stability. In addition, the direction of the CNF axis is vertical to the graphene nanosheets, which is able to control the diffusive orientation of the lithium ions.

**Figure 15.**
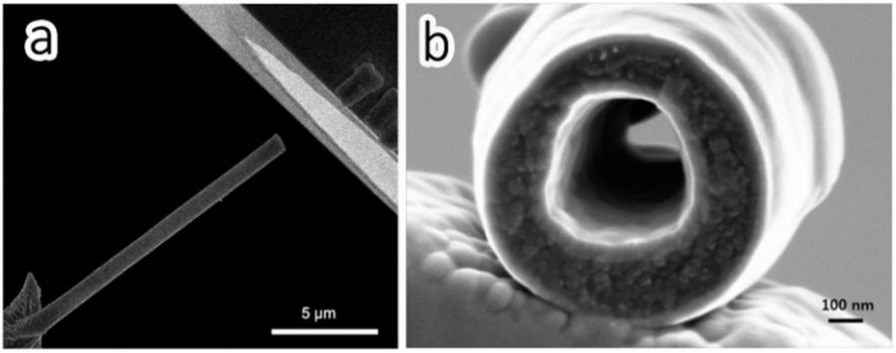
SEM images of (**a**) the fractured hollow CNF; and (**b**) the fractured surface of the hollow CNF [84].

Lee [84] found that the hollow CNFs used as freestanding electrodes in a flexible lithium ion battery are able to improve their mechanical properties by repeated electrochemical reactions with lithium ions. It was claimed that the inserted Li ions will form irreversible FCC crystals in the hollow CNF cavities and act as the reinforcement second phase of the carbonaceous matrix. Figure 15 shows the SEM image of the fractured hollow CNF.

**Figure 16.**
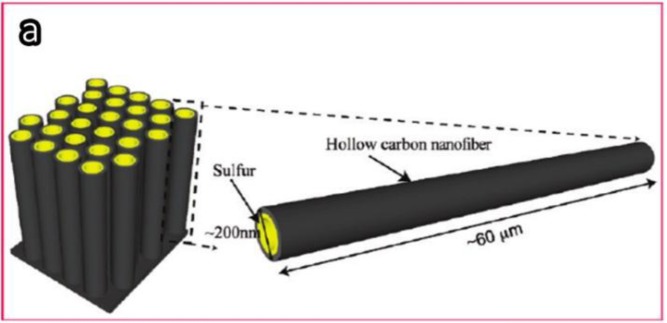

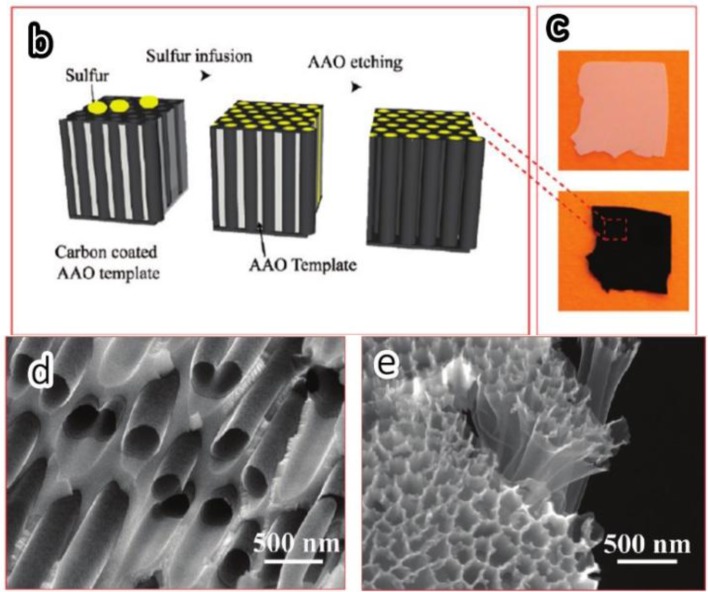
Schematic demonstration and SEM images of the hollow CNF/sulfur composite structure. (**a**) The high aspect ratio of the hollow CNF for the effective trapping of polysulfides; (**b**) the fabrication process of the CNF/sulfur cathode structure; (**c**) the contrast of the anodic aluminum oxide (AAO) template before and after carbon coating and sulfur infusion; (**d**) SEM image of the AAO template after carbon coating; and (**e**) hollow CNF encapsulated sulfur after etching away the AAO template [85].

Another hollow CNF structure was developed by Zheng [85]. In this study, the hollow CNF arrays were prepared through thermal carbonization of polystyrene by using anodic aluminum oxide as templates. Figure 16 gives the schematic illustration and SEM images of the hollow CNF/sulfur composite structure. A reversible capacity of about 730 mAh/g was achieved after 150 cycles. This structure successfully overcomes the shortcoming of the random diffusion of polysulfides in the organic electrolyte without sacrificing the fast transport of the lithium ions.

## 6. Conclusions and Future Perspectives

Generally, two preparation approaches, namely catalytic chemical vapor deposition growth and electrospinning, are the mainly effective pathways to fabricate CNFs. In the catalytic chemical vapor deposition growth method, some metals and alloys, including Fe, Co, Ni, Cr and V, which can dissolve carbon to form metal carbides, were able to be chosen as the catalysts, and the molybdenum, methane, carbon monoxide, synthesis gas (H_2_/CO), ethyne or ethane are able to be used as carbon sources. Generally, the structures of the CNF are decided by the shapes of the catalytic nano-sized metal particles. In the electrospinning process, the polymer types and the carbonization process play the most important roles in the quality of the prepared CNFs.

The overall properties of the CNF composites are largely governed by the dispersion condition of the CNFs in the matrix materials. To prepare the CNF composites with a good CNF dispersion, the melt mixing and solution process are the most widely used approaches. The melt mixing method, which was realized by high shear mixing, can effectively disperse the CNF in polymer matrix, while it is not able to guarantee the original aspect ratio and shapes of the CNFs. The solution process method, which is widely applied to disperse the CNF in a thermoset polymer matrix, is realized by sonication of CNFs in various solutions, followed by a curing process. In both of these methods, the chemical surface treatments of the CNFs are effective ways to realize their good dispersion in the matrix materials.

The electrical property of the CNF composites largely counts on the dispersion and percolation condition of the CNFs in the matrix. The percolation theory and fractal method are the decisive tools to evaluate the percolation threshold, the percolation backbone structure and the percolation critical exponents, which are the key factors to enhance the electrical properties of the CNF composites. Near the percolation threshold, most of the CNFs are not able to form a continuous network, and the dangling end parts are in the majority. The main electrical transport mechanism is the tunneling effect. Therefore, the surface treatment methods, the dispersion approaches and the polymer types are extremely important for the enhancement of the electrical properties. How to quantitatively determine the backbone structure of the CNFs in the matrix materials is still a challenging topic. The fractal analysis is an effective way of quantitatively characterizing the structure of the CNFs, and related models are waiting to be developed in future works to combine the overall performances and the microstructures of the CNF composites. In addition, the relationship between the thermal properties, the mechanical properties and the microstructures of the CNFs also need to be investigated more deeply, due to few studies demonstrating the effects of the CNF structures on the overall performance of the CNF composites.

As for applications, the CNFs and their composites are able to be used in many fields, including sensors, electrode materials and electromagnetic shielding. The sensitivity of the CNFs and their composites mainly count on their electrical performances. Therefore, how to accurately and quantitatively reflect the real situation by the electrical performances is the most important issue. Other than the vapor and strain/stress sensing, the humidity and temperature sensing capability of the CNFs and their composites are also waiting for development in future studies. As electrode materials, special structural designs and realization to guarantee high specific areas without satisfying mechanical performances is the key factor to enhancing the performances of the current materials.
